# Pre-exposure prophylaxis among men who have sex with men in the Amsterdam Cohort Studies: Use, eligibility, and intention to use

**DOI:** 10.1371/journal.pone.0205663

**Published:** 2018-10-12

**Authors:** Liza Coyer, Ward van Bilsen, Janneke Bil, Udi Davidovich, Elske Hoornenborg, Maria Prins, Amy Matser

**Affiliations:** 1 Department of Infectious Diseases, Research and Prevention, Public Health Service of Amsterdam, Amsterdam, the Netherlands; 2 Department of Infectious Diseases, Clinic for Sexually Transmitted Infections, Public Health Service of Amsterdam, Amsterdam, the Netherlands; 3 Department of Infectious Diseases, Amsterdam Infection & Immunity Institute (AIII), Amsterdam UMC, University of Amsterdam, Amsterdam, the Netherlands; Agencia de Salut Publica de Barcelona, SPAIN

## Abstract

**Objective:**

Currently, HIV pre-exposure prophylaxis (PrEP) is not covered by health insurance in the Netherlands. We examined time trends in use of PrEP, characteristics of PrEP users, PrEP eligibility and intention to use PrEP among HIV-negative men who have sex with men (MSM) participating in the Amsterdam Cohort Studies (ACS).

**Design:**

Prospective cohort study.

**Methods:**

We used data from four 6-monthly questionnaire waves, collected between 2015–2017. PrEP use over time was examined in logistic regression models using generalized estimating equations. Using descriptive statistics, we compared PrEP users before first-time initiation to non-PrEP-users. We used national guidelines to assess PrEP eligibility.

**Results:**

We included 687 MSM. Median age was 40 (IQR 33–47) years in 2015. Recent PrEP use was reported by 57/687 (8%) MSM. PrEP use increased over calendar time (*P*<0.001) to 7% in 2017. PrEP users did not differ from non-PrEP users in socio-demographic characteristics, but reported a significantly higher median number of casual sex partners, more often reported condomless anal sex and chemsex with casual partners, and more often had an sexually transmitted infection in the preceding 6 months (all *P*<0.05). PrEP eligibility increased over time, but the effect was not statistically significant (*P* = 0.075). PrEP eligibility criteria were met by 149/460 (32%) at wave 4, of whom 31/149 (21%) reported use of PrEP. The proportion with a high intention to use PrEP was greater among eligible than non-eligible MSM (51% vs. 24%, *P*<0.001).

**Conclusion:**

PrEP use increased over time but remained under 10%, even though 32% met the eligibility criteria, of whom 51% had a high intention to use PrEP. This suggests that a large proportion of Dutch MSM at risk could benefit from PrEP.

## Introduction

Despite proven efficacy and approval of pre-exposure prophylaxis (PrEP) for HIV prevention among men who have sex with men (MSM) in Europe, PrEP is not yet covered by health insurance in most countries, including the Netherlands [1,2]. In the Netherlands, MSM at high risk for HIV currently obtain PrEP free-of-charge only if participating in one of the two PrEP studies at the Public Health Service of Amsterdam: the Amsterdam PrEP project (AMPrEP), a demonstration project, initiated in 2015, to assess the uptake of daily and event-driven PrEP among HIV-negative MSM and transgender persons at increased risk for HIV infection [3], and the international multicenter DISCOVER study, which started enrolment in Amsterdam in 2017 and is aimed at evaluating the efficacy and safety of emtricitabine and tenofovir alafenamide for PrEP [4]. Alternatively, Dutch MSM can obtain PrEP by out-of-pocket purchase abroad or online or through friends or on a doctor’s prescription, which became legal after emtricitabine/tenofovir disoproxil was approved by the European Medicines Agency for the use of PrEP in July 2016 [5]. The price of 30 tablets decreased from 550 euro for the brand to around 50 euro for generics, which have been available since the beginning of 2018 [6]. A community-initiative of gay men has been informing MSM about PrEP access since 2015. This initiative organized self-importation and support for self-obtaining PrEP from the beginning of 2017 up to the availability of the generic product.

PrEP-related additional care (e.g. renal function and hepatitis C virus testing) is not routinely offered at sexually transmitted infections (STI) clinics in the Netherlands and capacity for quarterly visits, as is advised in professional guidelines, is limited. Although primary care providers can offer full testing, knowledge and willingness to prescribe PrEP varies across providers. Moreover, Dutch insurance systems have an obligatory deductible excess, meaning that a minimum of the first 385 euro of health care costs, including laboratory testing ordered by primary care providers, are out-of-pocket. This might cause reticence to visit a primary care provider.

Political support for PrEP has been patchy, with only some cities providing financial resources for health care for self-obtainers. In October 2016, the Minister of Health requested an advice on implementation of PrEP from the Health Council. The report was published in March 2018 and stated that the high burden of the HIV epidemic justifies implementation of PrEP, that a fee for the users can be considered, and that adequate monitoring of those who use PrEP is essential, in addition to national surveillance [7]. In July 2018, the Minister of Health decided to partially reimburse PrEP for 6,500 MSM at substantial risk for HIV within a research setting for a period of 5 years from 2019 onwards [8].

To further inform decision-making on full PrEP implementation and reimbursement, as well as resources needed, in the Netherlands, scientific data are needed on the number of MSM who would be eligible for PrEP, the current intention to use PrEP among MSM, and the characteristics of MSM who self-obtain PrEP [9].

The Amsterdam Cohort Studies (ACS) on HIV was initiated in 1984, and is an open, prospective cohort study among MSM [10]. PrEP awareness and the intention to use PrEP among MSM participating in the ACS was previously investigated between 2012 and 2013 [11]. In that period, 54% reported being aware of PrEP, but only 13% reported a high intention to use daily PrEP. In more recent years, PrEP awareness has likely increased. The added option of event-driven use of PrEP (i.e. PrEP use before and after sex) may have resulted in a higher PrEP uptake and intention to use PrEP. In this current study, we determined PrEP eligibility and intention to use PrEP among MSM participating in the ACS between 2015 and 2017. We moreover tracked PrEP use over time in the ACS and explored differences in characteristics between PrEP users and non-PrEP users.

## Materials and methods

### Study participants and data collection

HIV-negative MSM participating in the ACS provided data on PrEP through self-administered questionnaires in four 6-month waves from mid-2015 to mid-2017. At each ACS visit, we collected data on socio-demographic characteristics, sexual risk behavior, recreational drug use, chemsex, recent (i.e. in preceding 6 months) and lifetime PrEP use (S1 File); we also tested for HIV and STI (chlamydia, gonorrhea, syphilis) [10]. We distinguished use of study-provided PrEP (via AMPrEP/DISCOVER studies [4,12]) from use of self-obtained PrEP. Chemsex was defined as the use of γ-hydroxybutyric acid(GHB)/γ-butyrolactone (GBL), mephedrone and/or methamphetamine [13]. during sex with a casual partner.

PrEP eligibility for every participant was determined at each wave, using national guidelines provided by the Dutch Association of HIV-treating physicians [14]. PrEP eligibility criteria were 1) having had condomless anal sex (CAS) with a partner with unknown or seropositive HIV status, 2) having a rectal chlamydia or gonorrhea, and/or 3) having a post-exposure prophylaxis (PEP) prescription, all reported or diagnosed in the preceding six months.

Intention to use PrEP was measured at wave 1, separately for daily and event-driven use, by two questions on a seven-point Likert scale: “How likely are you to use PrEP once it becomes available in the Netherlands?” and “Are you planning on using PrEP once it becomes available in the Netherlands?” (S1 File). A score of >4, the median of all MSM, was defined as a high intention for either daily or event-driven PrEP use. A score of <2 was defined as low intention for PrEP use and a score of 2–4 as medium intention for PrEP use, as described previously by Bil et al. [11]. If the score differed between daily and event-driven use, we used the highest one.

### Statistical analysis

To compare socio-demographic characteristics, sexual behavior, recreational drug use, chemsex, STI diagnosis, and PrEP eligibility in the six months prior to PrEP initiation between PrEP users (those reporting use at least once during waves 1–4) and non-PrEP users (those never reporting PrEP use), we employed the unpaired *t*-test for normally distributed numerical data, the Mann-Whitney *U* test for non-normally distributed numerical data, and the Chi-square or Fisher’s exact test for categorical data. MSM who reported PrEP use only before 2015 were excluded for this analysis. Information on characteristics and behaviors of PrEP users was taken from the wave prior to the one at which first-time PrEP was reported; information on non-PrEP users was taken from the wave at which most participants reported first-time PrEP use (wave 2). We performed three sensitivity analyses in which we compared PrEP users to non-PrEP users, taking information of non-PrEP users from wave 1, 3 and 4. Time trends in PrEP use and PrEP eligibility were examined in logistic regression models using generalized estimating equations to account for clustering within individuals. The effect of missing data on the PrEP eligibility estimate was explored in sensitivity analyses, assuming extreme values for missing data.

Results were considered significant at a p-value ≤0.05. Analyses were performed using STATA Intercooled 13.1 (STATA Corporation, College Station, Texas, USA).

### Ethical approval and informed consent

The ACS was approved by the Medical Ethical Committee of the Academic Medical Center of Amsterdam, the Netherlands. Written informed consent was obtained from all participants at enrolment.

## Results

Our study included 687 HIV-negative MSM, of whom the majority was educated at least to college degree (n = 529, 77%) and born in the Netherlands (n = 545, 79%). Median age was 40 (IQR 33–47) years at wave 1 of the study period. Median number of questionnaire waves per participant was 4 (IQR 3–4).

### Use of PrEP and characteristics of PrEP users

**Fig 1:**
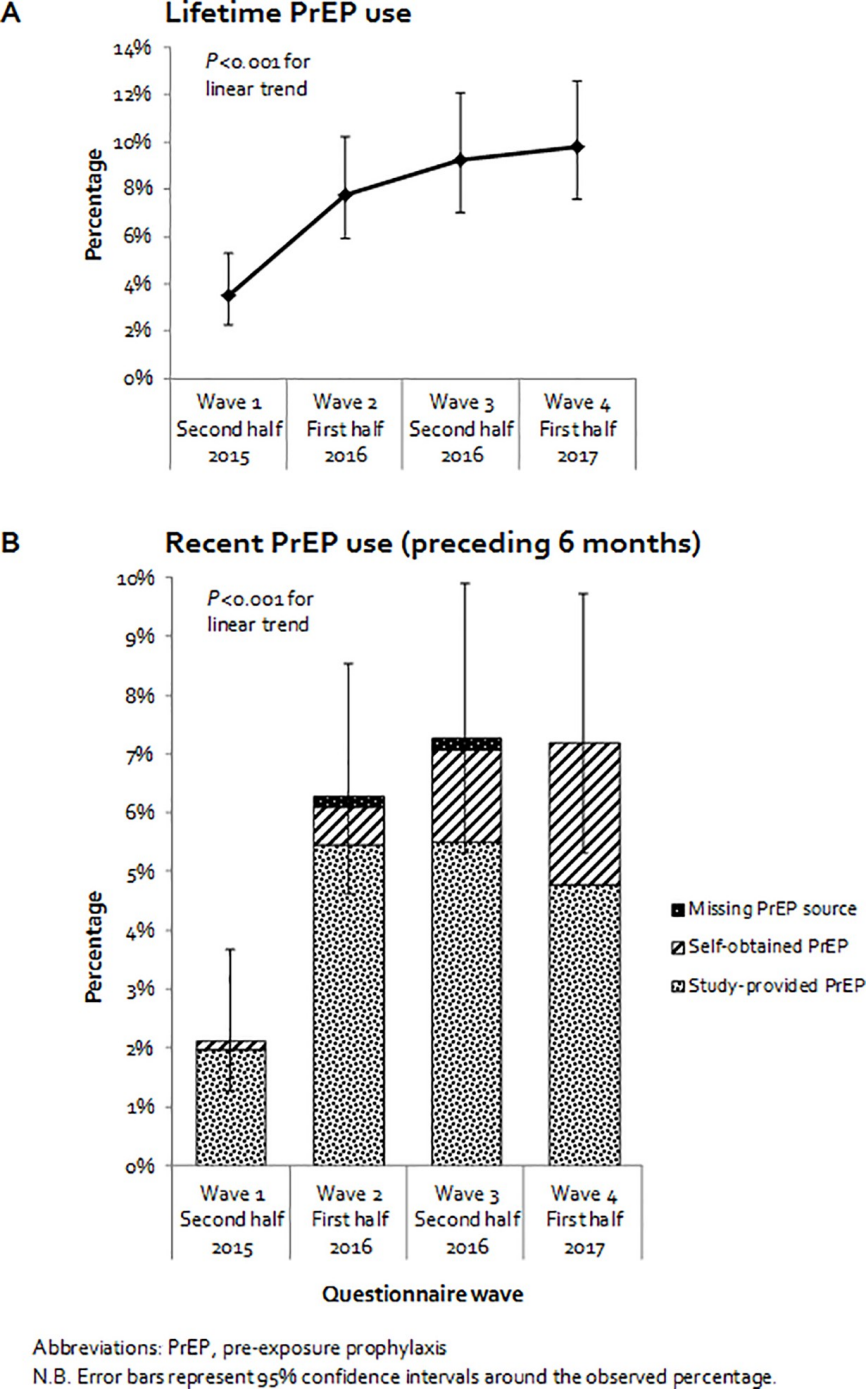
Reported lifetime and recent PrEP use among MSM participating in the Amsterdam Cohort Studies between 2015–2017 (four 6-montly waves of questionnaires).

Recent PrEP use was reported by 57 (8%; 95% confidence interval [CI] 6–11%), whereas 621 (90%) reported no PrEP use, and 9 (1%) only reported use before 2015. Of the 57 users, 38 (67%) used study-provided PrEP, and 19 (33%) used self-obtained PrEP at first-time initiation. As for regimen, 20 (35%) reported daily use, 22 (39%) reported event-driven use, and 15 (26%) had missing information on regimen at first-time initiation. Median age at first-time initiation was 40 (IQR 36–48) years. PrEP use increased from 2015 to 2017, with both recent and lifetime use showing linear trends (*P*<0.001, Fig 1).

Behavioral data were available for 52/57 PrEP users and 541/621 non-users. Users and non-users did not differ in age, country of birth, or educational level (Table 1). PrEP users had a significantly higher median number of casual partners compared to non-PrEP users (21 [IQR 12–40] vs. 4 [IQR 0–11], *P*<0.001), and were more likely to report chemsex (43% vs. 7%, *P*<0.001) and CAS (85% vs. 26%, p<0.001) with casual partners. STIs were more often diagnosed among PrEP users than non-PrEP users (27% vs. 11%, *P* = 0.001), mainly rectal chlamydia (13% vs. 4%, p = 0.005) and syphilis (8% vs. 1%, *P* = 0.008). No differences were found in having a steady partner or having CAS with a steady partner (all *P*>0.05). Of 48 PrEP users with available eligibility information, 34 (71%) were eligible for PrEP, compared to 118/493 (24%) non-PrEP users (*P*<0.001). Of 16 users of self-obtained PrEP, 10 (63%) were eligible for PrEP prior to initiation. These results were comparable to the results of the sensitivity analyses using information on non-PrEP users of wave 1, 3, and 4.

**Table 1:** Characteristics of MSM participating in the Amsterdam Cohort Studies between 2015 and 2017: PrEP users before first-time PrEP initiation versus non-PrEP-users.

	PrEP users		Non-PrEP-users		
	(n = 52)		(n = 541)		
Socio-demographic characteristics	N	n (%)	N	n (%)	*P-*value
Age in years (median, IQR)	52	40 [36–48]	541	41 [34–48]	0.459
Born in the Netherlands	49	43 (88)	512	437 (85)	0.647
High education level (college degree or higher)	52	41 (79)	541	422 (78)	0.888
**Sexual behavior**					
Steady partner(s) in preceding 6 months					
≥1 Steady partner	48	29 (60)	519	327 (63)	0.723
Anal sex with steady partner	48	21 (44)	519	238 (46)	0.966
CAS with steady partner(s)	48	20 (42)	519	200 (39)	0.670
CAS with HIV+ steady partner or partner with unknown HIV status	48	3 (6)	508	2(5)	0.687
Casual partner(s) in preceding 6 months					
≥1 Casual partner	48	48 (100)	514	357 (69)	<0.001
Number of casual partner(s) (median, IQR)	48	21 [12–40]	514	4 [0–11]	<0.001
Anal sex with casual partner	48	48 (100)	512	297 (58)	<0.001
CAS with casual partner(s)	48	41 (85)	511	134 (26)	<0.001
CAS with HIV+ casual partner(s) or partner with unknown HIV status	48	31 (64)	511	76 (15)	<0.001
**Recreational drug use**					
Any illicit drug use* in preceding 6 months	47	27 (57)	517	197 (38)	0.009
Chemsex** with casual partners in preceding 6 months	47	20 (43)	517	37 (7)	<0.001
Injecting drug use in preceding 6 months	47	0	518	0	-
**STI diagnosis in preceding 6 months**					
Any bacterial STI	52	14 (27)	527	60 (11)	0.001
Any rectal STI (chlamydia/gonorrhea)	52	8 (15)	527	35 (7)	0.022
Chlamydia, urethral	52	2 (4)	541	10 (2)	0.347
Chlamydia, rectal	52	7 (13)	527	23 (4)	0.005
Gonorrhea, urethral	52	2 (4)	527	7 (1)	0.190
Gonorrhea, rectal	52	4 (8)	527	16 (3)	0.079
Syphilis	52	4 (8)	527	6 (1)	0.008
**PrEP eligibility and criteria**					
Eligible for PrEP in the preceding 6 months	48	34 (71)	493	118 (24)	<0.001
CAS with partner with unknown or seropositive HIV status	48	31 (60)	501	96 (19)	<0.001
Rectal chlamydia or gonorrhea	52	8 (15)	527	35 (7)	0.022
PEP prescription	49	4 (8)	539	7 (1)	0.001
**Intention to use PrEP**					
High intention to use PrEP in 2015 (vs. low/medium)	45	36 (80)	455	119 (26)	<0.001
High intention for daily use (vs. low/medium)	45	25 (56)	455	65 (14)	<0.001
High intention for event-driven use (vs. low/medium)	45	25 (56)	455	84 (18)	<0.001

Abbreviations: CAS, condomless anal sex; HIV, human immunodeficiency virus; IQR, interquartile range; MSM, men who have sex with men; STI, sexually transmitted infection; PrEP, pre-exposure prophylaxis; PEP, post-exposure prophylaxis.

*Defined as the use of amphetamines, benzodiazepines, cocaine, 2,5-dimethoxy-4-bromophenethylamine (2-CB), 4-Fluoroamphetamine (4-FA), γ-hydroxybutyric acid(GHB)/γ-butyrolactone (GBL), heroin, ketamin, mephedrone, methamphetamin, opioids, 3,4-methylenedioxymethamphetamine (XTC/MDMA), 3-mmc, methoxetamin (MXE), 4-methylethcathinone (4-mec).

**Defined as the use of GHB/GBL, mephedrone and/or methamphetamine [13] during sex with a casual partner.’

### PrEP eligibility and intention to use PrEP

**Fig 2:**
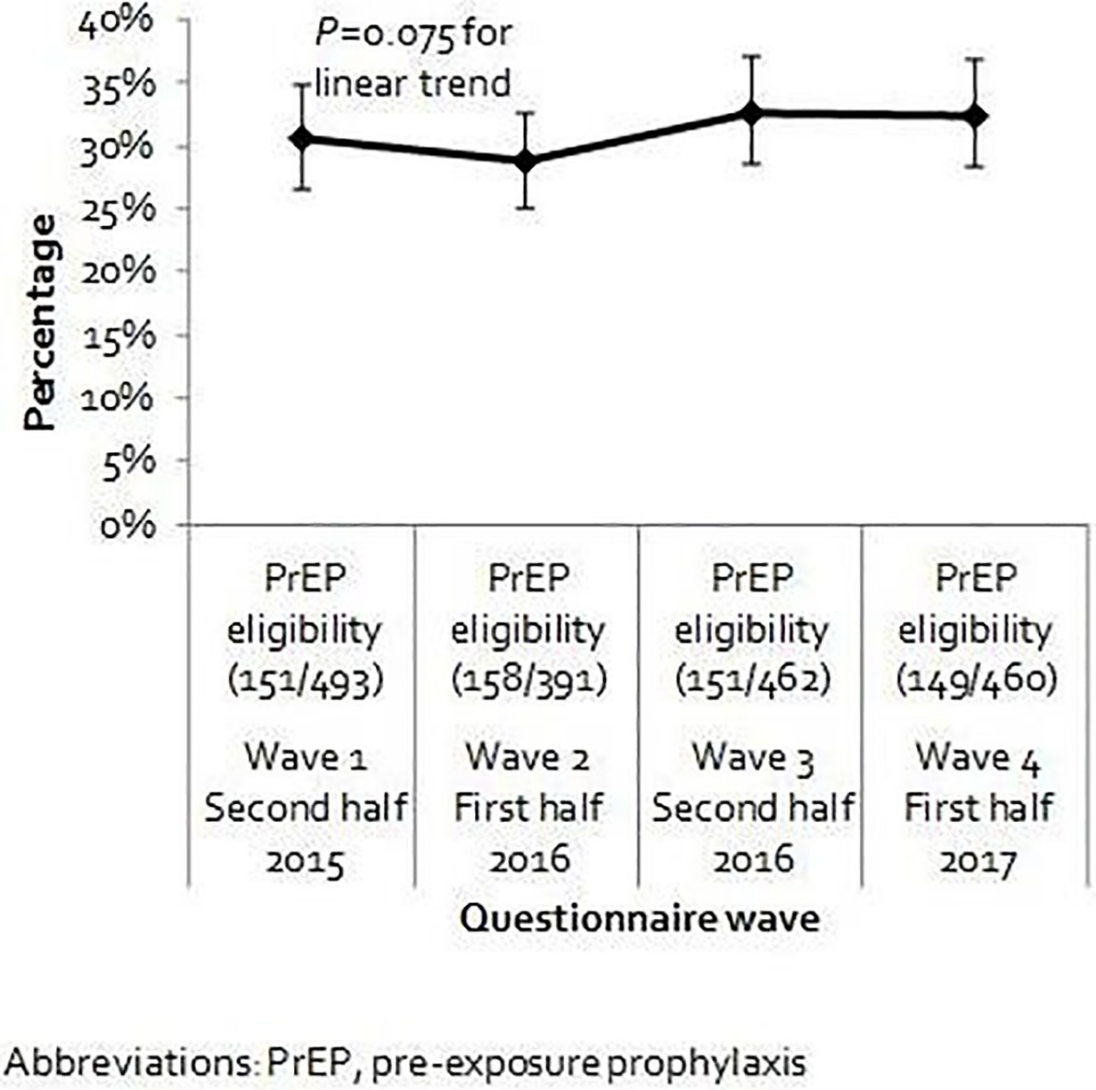
PrEP eligibility among MSM participating in the Amsterdam Cohort Studies between 2015–2017.

We observed a small increase in PrEP eligibility from 2015 to 2017, but the effect was not statistically significant (*P* = 0.075, Fig 2). Of 460 MSM with eligibility information available at wave 4, 149 (32%, 95% CI 28–37%) met ≥1 PrEP eligibility criteria; of these, 31/149 (21%) reported lifetime PrEP use. Among eligible MSM, the majority (n = 127/149, 85%) reported CAS with a partner with positive (n = 29) and/or unknown HIV serostatus (n = 103). In total, 21/149 (14%) MSM were considered eligible for PrEP based only on reported CAS with an HIV-positive partner. Thirty-nine of 149 (26%) MSM had a rectal chlamydia or gonorrhea diagnosis in the preceding 6 months. PEP, the third PrEP eligibility criterion, had been prescribed to 6/149 (4%) MSM. In our sensitivity analysis on missing eligibility data, PrEP eligibility at wave 4 was 27% (n = 149/541, 95% CI 24–31%) when MSM with missing data (81/541, 15%) were considered not eligible, whereas it was 43% (230/541, 95% CI 38–47%) when all were considered eligible.

Of 548 MSM with data on intention to use PrEP, 165 (30% [95% CI 26–34%]) reported a high intention, 277 (51% [95% CI 46–55%]) a medium intention and 106 (19% [95% CI 16–23%]) a low intention. The proportion with a high intention was greater among eligible compared to non-eligible MSM (51% [95% CI 43–59%] vs. 24% [95% CI 20–29%], p<0.001), and also greater among MSM who initiated PrEP between 2015 and 2017 compared to MSM who did not (80% vs. 26%, *P*<0.001). A high intention for daily use was reported by 96 (17%) MSM, and for event-driven use by 114 (21%) MSM.

## Discussion

Approximately 10% of HIV-negative participants of a prospective MSM cohort in Amsterdam, the Netherlands, reported lifetime PrEP use through the first half of 2017. The number of PrEP users had increased since 2015 and most obtained PrEP through participation in studies. PrEP users did not differ from non-PrEP users in terms of socio-demographic characteristics. According to national guidelines, about 30% were eligible for PrEP at the most recent wave, of whom about half had a high intention to use PrEP and one-fifth had ever used PrEP.

This study is subject to some limitations. First, ACS participants were predominantly highly educated native Dutch MSM from the Amsterdam area who may not represent nationwide MSM. They are likely to be more informed about PrEP, given the close proximity of ongoing PrEP studies at the Public Health Service of Amsterdam. Second, not all MSM with a study visit at wave 4 had complete eligibility information available. Sensitivity analysis showed that in extreme-value scenarios, eligibility ranged between 27% and 43%. Third, our eligibility criterion concerning CAS with a partner with positive or unknown HIV status did not include the undetectable viral load criterion specified in the national guidelines, as data was not available. We may therefore have misclassified some of the 21/149 MSM who were eligible based only on reported CAS with an HIV-positive partner, as eligible. Finally, the differences in sexual risk behavior and STI between PrEP users and non-users likely reflect the eligibility criteria of the studies through which most users in this cohort obtained PrEP. The low number of PrEP users did not permit subgroup analyses to compare MSM using PrEP from various sources or those following different dosing regimens.

Estimated numbers of PrEP users and PrEP-eligible persons in Europe are scarce and mainly based on personal communications [2]. Estimations range from 500–1500 eligible persons in Belgium to 20,000–100,000 in England [2]. To our knowledge, our study provides the first estimates of PrEP eligibility and intention to use among MSM in the Netherlands. These findings can be used to provide crude estimates of the number of indicated and expected PrEP users, and associated costs, in the absence of more nationally representative data. Based on our findings, the estimated total number of MSM in Amsterdam [15], and the HIV prevalence among MSM [16], we roughly estimate that between 2,903–5,370 eligible MSM in Amsterdam can be expected to use PrEP (S2 File). This is higher than a previous estimate using STI clinic data and the ACS data from 2012 (n = 936–2,358) [11,17]. Additionally, between 2,986–5,118 non-eligible MSM may also use PrEP (S2 File). These estimates may be slightly overestimated as PrEP use among ACS participants might be somewhat ahead of PrEP use in the general MSM population due to having been informed about PrEP earlier by study participation. Moreover, we used intention as a marker of uptake, but intention does not fully predict use, and uptake depends on multiple factors [18].

The percentage of MSM with a high intention to use PrEP has more than doubled since a 2012 study in the same cohort [11], possibly due to increased PrEP awareness, new results on PrEP effectiveness [19,20], and the added option of event-driven PrEP use. The increased PrEP awareness might have been influenced by long-term study participation, but also by an increased information provision on PrEP to MSM in the Netherlands which has been going on since 2015. The proportion with a high intention to use daily PrEP was smaller (17%) but was still increased compared to 2012. Since about half of eligible MSM did not report a high intention, future work is warranted on how to motivate MSM at risk of HIV to use PrEP. As observed in the 2012 study, this includes increasing PrEP knowledge; addressing psychosocial determinants such as feelings of shame and perception of self-efficacy; and encouraging the use of condoms or other risk reduction strategies alongside increasing PrEP accessibility [11, 21]. We furthermore expect PrEP intention and use to increase with increasing awareness and decreasing PrEP costs [10,20–22]. This is supported by the observed increasing proportion of recent self-obtaining PrEP users.

In conclusion, this study provides important descriptive data for decision-making on PrEP implementation in the Netherlands. The majority of eligible MSM are currently not using PrEP, suggesting that a large proportion of MSM at risk for HIV could benefit from it once accessibility improves. As accessibility improves, there should be a focus on improving intention to use PrEP among eligible MSM who do not have a high intention to use PrEP. Research on motives for and barriers to using PrEP can be helpful to increase PrEP uptake among eligible MSM.

## Supporting information

S1 File Survey questions. (PDF)

S2 File Calculation of the estimated number of expected PrEP users in Amsterdam, the Netherlands. (PDF)
