# Supplementary material for: Pre-exposure prophylaxis among men who have sex with men in the Amsterdam Cohort Studies: Use, eligibility, and intention to use
Source: PLoS One. 2018 Oct 12;13(10):e0205663. doi: 10.1371/journal.pone.0205663 (PMC6185853; doi:10.1371/journal.pone.0205663)
Supplement: S1 File — (PDF) [file pone.0205663.s001.pdf]

Supplement 1 File. Survey questions

Questions on steady relationships, sex with casual partners and drugs (English)
Questionnaire waves 1-4

1. Did you have one or more steady relationships with a male in the preceding 6 months?
   ☐ No
   ☐ Yes, with _______ men

2. Do you know the HIV-status of your steady partner?
   ☐ No
   ☐ Yes

   Is this information based on his latest HIV-test?
   ☐ Yes, how long ago was your partner tested?

   ☐ ±1 month ☐ ±3 months ☐ ±6 months
   ☐ ±1 year ☐ ±2 years or longer ☐ I don't know

   The result was:
   ☐ seronegative
   ☐ seropositive

   ☐ No, the information is not based on an HIV test but on the fact that:

   ☐ My partner told me he was HIV negative
   ☐ My partner told me to be a virgin or that he was not at risk for HIV
   ☐ I trust my partner
   ☐ Otherwise, namely _________________________________

3. Did you fuck your steady partner?
   ☐ No
   ☐ Yes

   How often did you use a condom?
   ☐ Never
   ☐ Most of the times not
   ☐ Sometimes I did – sometimes I didn't
   ☐ Most of the times
   ☐ Always

4. Did you get fucked by your steady partner?
   ☐ No
   ☐ Yes

   How often did you use a condom?
   ☐ Never
   ☐ Most of the times not
   ☐ Sometimes I did – sometimes I didn't
   ☐ Most of the times
   ☐ Always

5. Did you fuck your casual partner(s)?
   ☐ No
   ☐ Yes
   Namely:

   With one-time casual partners?
   ☐ No
   ☐ Yes, with ___ one-time casual partner(s)

   With multiple-time casual partners?
   ☐ No
   ☐ Yes, with ___ multiple-time casual partner(s)

   With regular casual partners (fuck buddies)?
   ☐ No
   ☐ Yes, with ___ regular casual partners (fuck buddies)

How often did you use a condom?
☐ Never
☐ Most of the times not
☐ Sometimes I did – sometimes I didn't
☐ Most of the times
☐ Always

How often did you use a condom?
☐ Never
☐ Most of the times not
☐ Sometimes I did – sometimes I didn't
☐ Most of the times
☐ Always

How often did you use a condom?
☐ Never
☐ Most of the times not
☐ Sometimes I did – sometimes I didn't
☐ Most of the times
☐ Always

6. Did you fuck a casual partner of whom you did not know the HIV status without a condom?
☐ No
☐ Yes
Namely:

By one-time casual partners?
☐ No
☐ Yes, with ___ one-time casual partner(s)

By multiple-time casual partners?
☐ No
☐ Yes, with ___ multiple-time casual partner(s)

By regular casual partners (fuck buddies)?
☐ No
☐ Yes, with ___ regular casual partners (fuck buddies)

7. Did you fuck a HIV positive casual partner without a condom?
☐ No
☐ Yes
Namely:

With one-time casual partners?
☐ No
☐ Yes, with ___ one-time casual partner(s)

With multiple-time casual partners?
☐ No
☐ Yes, with ___ multiple-time casual partner(s)

With regular casual partners (fuck buddies)?
☐ No
☐ Yes, with ___ regular casual partners (fuck buddies)

8. Did you get fucked by casual partners?
☐ No
☐ Yes
Namely:

By one-time casual partners?
☐ No
☐ Yes, with ___ one-time casual partner(s)

By multiple-time casual partners?
☐ No
☐ Yes, with ___ multiple-time casual partner(s)

By regular casual partners (fuck buddies)?
☐ No
☐ Yes, with ___ regular casual partners (fuck buddies)

How often did you use a condom?
☐ Never
☐ Most of the times not
☐ Sometimes I did – sometimes I didn't
☐ Most of the times
☐ Always

How often did you use a condom?
☐ Never
☐ Most of the times not
☐ Sometimes I did – sometimes I didn't
☐ Most of the times
☐ Always

How often did you use a condom?
☐ Never
☐ Most of the times not
☐ Sometimes I did – sometimes I didn't
☐ Most of the times
☐ Always

9. Did you get fucked by casual partners of whom you did not know the HIV status without a condom?
☐ No
☐ Yes
Namely:

By one-time casual partners?
☐ No
☐ Yes, with ___ one-time casual partner(s)

By multiple-time casual partners?
☐ No
☐ Yes, with ___ multiple-time casual partner(s)

By regular casual partners (fuck buddies)?
☐ No
☐ Yes, with ___ regular casual partners (fuck buddies)

10. Did you get fucked by HIV positive casual partners without a condom?
☐ No
☐ Yes
Namely:

By one-time casual partners?
☐ No

By multiple-time casual partners?
☐ No

By regular casual partners (fuck buddies)?

	☐ Yes, with ___ one-time casual partner(s)	☐ Yes, with ___ multiple-time casual partner(s)	☐ No<br>☐ Yes, with ___ regular casual partners (fuck buddies)

11. Did you use any of the following in the preceding 6 months?
If yes, in which context and how? (Multiple answers possible)

	In general		During sex with:				
		If yes, how often?	Steady partner:	One-time casual partner:	Multiple-time casual partner:	Regular casual partner:	Groupsex:
Alcohol	☐ no	☐ 1x per month or less<br>☐ 2-4x per month<br>☐ 2-3x per week<br>☐ 4x per week or more	☐ yes	☐ yes	☐ yes	☐ yes	☐ yes
Nitrites	☐ no	☐ 1x per month or less<br>☐ 2-4x per month<br>☐ 2-3x per week<br>☐ 4x per week or more	☐ yes	☐ yes	☐ yes	☐ yes	☐ yes
XTC/MDMA	☐ no	☐ 1x per month or less<br>☐ 2-4x per month<br>☐ 2-3x per week<br>☐ 4x per week or more	☐ yes	☐ yes	☐ yes	☐ yes	☐ yes
Erectile dysfunction drugs	☐ no	☐ 1x per month or less<br>☐ 2-4x per month<br>☐ 2-3x per week<br>☐ 4x per week or more	☐ yes	☐ yes	☐ yes	☐ yes	☐ yes
Hash/Weed	☐ no	☐ 1x per month or less<br>☐ 2-4x per month<br>☐ 2-3x per week<br>☐ 4x per week or more	☐ yes	☐ yes	☐ yes	☐ yes	☐ yes
Cocaine	☐ no	☐ 1x per month or less<br>☐ 2-4x per month<br>☐ 2-3x per week<br>☐ 4x per week or more	☐ yes	☐ yes	☐ yes	☐ yes	☐ yes
Mephedrone	☐ no	☐ 1x per month or less<br>☐ 2-4x per month<br>☐ 2-3x per week<br>☐ 4x per week or more	☐ yes	☐ yes	☐ yes	☐ yes	☐ yes
GHB	☐ no	☐ 1x per month or less<br>☐ 2-4x per month<br>☐ 2-3x per week<br>☐ 4x per week or more	☐ yes	☐ yes	☐ yes	☐ yes	☐ yes
GBL	☐ no	☐ 1x per month or less<br>☐ 2-4x per month<br>☐ 2-3x per week<br>☐ 4x per week or more	☐ yes	☐ yes	☐ yes	☐ yes	☐ yes
Ketamine	☐ no	☐ 1x per month or less<br>☐ 2-4x per month<br>☐ 2-3x per week<br>☐ 4x per week or more	☐ yes	☐ yes	☐ yes	☐ yes	☐ yes
Mushrooms	☐ no	☐ 1x per month or less<br>☐ 2-4x per month<br>☐ 2-3x per week<br>☐ 4x per week or more	☐ yes	☐ yes	☐ yes	☐ yes	☐ yes
Amphetamine	☐ no	☐ 1x per month or less<br>☐ 2-4x per month<br>☐ 2-3x per week<br>☐ 4x per week or more	☐ yes	☐ yes	☐ yes	☐ yes	☐ yes
Methylamphetamine	☐ no	☐ 1x per month or less<br>☐ 2-4x per month<br>☐ 2-3x per week<br>☐ 4x per week or more	☐ yes	☐ yes	☐ yes	☐ yes	☐ yes

	**In general**		**During sex with:**				
	**If yes, how often?**		**Steady partner:**	**If yes, how often?**	**Steady partner:**	**If yes, how often?**	**Steady partner:**
Heroin	☐ no	☐ 1x per month or less<br>☐ 2-4x per month<br>☐ 2-3x per week<br>☐ 4x per week or more	☐ yes	☐ yes	☐ yes	☐ yes	☐ yes
Apomorphine	☐ no	☐ 1x per month or less<br>☐ 2-4x per month<br>☐ 2-3x per week<br>☐ 4x per week or more	☐ yes	☐ yes	☐ yes	☐ yes	☐ yes
Testosterone	☐ no	☐ 1x per month or less<br>☐ 2-4x per month<br>☐ 2-3x per week<br>☐ 4x per week or more	☐ yes	☐ yes	☐ yes	☐ yes	☐ yes
Anabolic steroids	☐ no	☐ 1x per month or less<br>☐ 2-4x per month<br>☐ 2-3x per week<br>☐ 4x per week or more	☐ yes	☐ yes	☐ yes	☐ yes	☐ yes
DHEA	☐ no	☐ 1x per month or less<br>☐ 2-4x per month<br>☐ 2-3x per week<br>☐ 4x per week or more	☐ yes	☐ yes	☐ yes	☐ yes	☐ yes
4-FA/4-FMP	☐ no	☐ 1x per month or less<br>☐ 2-4x per month<br>☐ 2-3x per week<br>☐ 4x per week or more	☐ yes	☐ yes	☐ yes	☐ yes	☐ yes
2-CB	☐ no	☐ 1x per month or less<br>☐ 2-4x per month<br>☐ 2-3x per week<br>☐ 4x per week or more	☐ yes	☐ yes	☐ yes	☐ yes	☐ yes
Antidepressants (not as medication)	☐ no	☐ 1x per month or less<br>☐ 2-4x per month<br>☐ 2-3x per week<br>☐ 4x per week or more	☐ yes	☐ yes	☐ yes	☐ yes	☐ yes
Methylphenidate (not as medication)	☐ no	☐ 1x per month or less<br>☐ 2-4x per month<br>☐ 2-3x per week<br>☐ 4x per week or more	☐ yes	☐ yes	☐ yes	☐ yes	☐ yes
Opiods (not as medication)	☐ no	☐ 1x per month or less<br>☐ 2-4x per month<br>☐ 2-3x per week<br>☐ 4x per week or more	☐ yes	☐ yes	☐ yes	☐ yes	☐ yes
Benzodiazepines (not as medication)	☐ no	☐ 1x per month or less<br>☐ 2-4x per month<br>☐ 2-3x per week<br>☐ 4x per week or more	☐ yes	☐ yes	☐ yes	☐ yes	☐ yes
Other, namely ____________________	☐ no	☐ 1x per month or less<br>☐ 2-4x per month<br>☐ 2-3x per week<br>☐ 4x per week or more	☐ yes	☐ yes	☐ yes	☐ yes	☐ yes

<u>Original questions on PrEP use and intention to use PrEP (English)</u>
Questionnaire wave 1

12. Have you have used PrEP?
    - ☐ No
    - ☐ Yes

13. Have you used daily PrEP in the preceding 6 months?
    - ☐ No
    - ☐ Yes

14. When did you start using daily PrEP? (In case you started daily PrEP multiple times, only fill in the last time you started): _ _ / _ _ _ _ (MM/JJJJ)

15. Do you currently still use daily PrEP?
    - ☐ No, I quite in _ _ / _ _ _ _ (MM/JJJJ)
    - ☐ Yes

16. Did you use event-driven PrEP in the preceding 6 months?
    - ☐ No
    - ☐ Yes

17. Where did you obtain your PrEP? (Multiple answers allowed)
    - ☐ Abroad
    - ☐ Via internet
    - ☐ Via friends
    - ☐ Via someone who is HIV positive
    - ☐ Via a doctor of the AmPrEP-project (STI clinic Amsterdam)
    - ☐ Via an HIV doctor in the hospital
    - ☐ Otherwise, namely: ___

18. a) Are you going to use daily PrEP once PrEP becomes available in the Netherlands?
    Absolutely not 1 2 3 4 5 6 7 Definitely
    b) How likely are you to use daily PrEP once PrEP becomes available in the Netherlands?
    Very likely 1 2 3 4 5 6 7 Very unlikely
    c) Are you going to use event-driven PrEP once PrEP becomes available in the Netherlands?
    Absolutely not 1 2 3 4 5 6 7 Definitely
    d) How likely are you to use event-driven PrEP once PrEP becomes available in the Netherlands?
    Verly likely 1 2 3 4 5 6 7 Very unlikely

19. Imagine you are going to use PrEP once it becomes available in the Netherlands. Which regimen would be most suitable for you? Assume both regimens are equally effective in preventing HIV in answering this question.
    - ☐ Daily PrEP
    - ☐ Event-driven PrEP

Original questions on PrEP use (English)
Questionnaire wave 2-4

20. Did you use PrEP in the preceding 6 months?
    - ☐ No
    - ☐ Yes

21. How did you use PrEP in the preceding 6 months?
    - ☐ I use(d) it daily
    - ☐ I use(d) it event-driven (event-driven means not daily taking PrEP, but for example only before and after sex in case there was a risk of getting HIV)

22. When did you start using daily PrEP? (In case you started daily PrEP multiple times, only fill in the last time you started): _ _ / _ _ _ _ (MM/JJJJ)

23. Do you currently still use daily PrEP?
    - ☐ No, I quite in _ _ / _ _ _ _ (MM/JJJJ)
    - ☐ Yes

24. Where did you obtain your PrEP? (Multiple answers allowed)
    - ☐ Abroad
    - ☐ Via internet
    - ☐ Via friends
    - ☐ Via someone who is HIV positive
    - ☐ I bought it from someone
    - ☐ I am a participant of the AmPrEP-project (STI clinic Amsterdam)
    - ☐ Via an HIV doctor in the hospital
    - ☐ Via another doctor who is not treating HIV
    - ☐ Otherwise, namely: ___

Questions on steady relationships, sex with casual partners and drugs (Dutch)
Questionnaire waves 1-4

1. Heeft u in de afgelopen 6 maanden (een) vaste relatie(s) gehad met een man?
   - ☐ Nee
   - ☐ Ja, met _______ mannen.

2. Weet u de HIV-status van uw vaste partner?
   - ☐ Nee
   - ☐ Ja

     Is die informatie gebaseerd op zijn laatste HIV-test?
     - ☐ Ja, hoe lang geleden is uw partner getest?

       ☐ ±1 Maand ☐ ±3 Maanden ☐ ±6 Maanden
       ☐ ±1 Jaar ☐ ±2 Jaar geleden of langer ☐ Ik weet het niet

       De uitslag was:
       - ☐ Seronegatief
       - ☐ Seropositief

     - ☐ Nee, de informatie is niet gebaseerd op een HIV test maar op:
       - ☐ Dat mijn partner gezegd heeft negatief te zijn.
       - ☐ Dat mijn partner verteld heeft maagd te zijn of geen risico te hebben gelopen.
       - ☐ Dat mijn partner mij niks verteld heeft, maar ik hem wel vertrouw.
       - ☐ Anders, nl.__________________________________

3. Heeft u uw vaste partner geneukt?
   - ☐ Nee
   - ☐ Ja

     Hoe vaak heeft u daarbij een condoom gebruikt?
     - ☐ Nooit
     - ☐ Meestal niet
     - ☐ Soms wel – soms niet
     - ☐ Meestal wel
     - ☐ Altijd

4. Heeft u zicht door uw vaste partner laten neuken?
   - ☐ Nee
   - ☐ Ja

     Hoe vaak heeft u daarbij een condoom gebruikt?
     - ☐ Nooit
     - ☐ Meestal niet
     - ☐ Soms wel – soms niet
     - ☐ Meestal wel
     - ☐ Altijd

5. Heeft u uw losse partners geneukt?
   - ☐ Nee
   - ☐ Ja

     Namelijk:

Met eenmalige losse partners?	Met meermalige losse partners?	Met vaste seks partners (seksmaatjes)?
☐ Nee	☐ Nee	☐ Nee
☐ Ja, met _____ eenmalige losse partner(s)	☐ Ja, met _____ meermalige losse partner(s)	☐ Ja, met _____ vaste seks partner(s) (seksmaatjes)

Hoe vaak heeft u daarbij een condoom gebruikt?	Hoe vaak heeft u daarbij een condoom gebruikt?	Hoe vaak heeft u daarbij een condoom gebruikt?
☐ Nooit	☐ Nooit	☐ Nooit
☐ Meestal niet	☐ Meestal niet	☐ Meestal niet
☐ Soms wel – soms niet	☐ Soms wel – soms niet	☐ Soms wel – soms niet
☐ Meestal wel	☐ Meestal wel	☐ Meestal wel
☐ Altijd	☐ Altijd	☐ Altijd

6. Heeft u een losse partner van wie u de HIV-status niet wist onbeschermd geneukt?
☐ Nee
☐ Ja
Namelijk:

Met eenmalige losse partners?	Met meermalige losse partners?	Met vaste seks partners (seksmaatjes)?
☐ Nee	☐ Nee	☐ Nee
☐ Ja, met _____ eenmalige losse partner(s)	☐ Ja, met _____ meermalige losse partner(s)	☐ Ja, met _____ vaste seks partner(s) (seksmaatjes)

7. Heeft u een losse partner van wie u al wist dat hij HIV-geïnfecteerd was onbeschermd geneukt?
☐ Nee
☐ Ja
Namelijk:

Met eenmalige losse partners?	Met meermalige losse partners?	Met vaste seks partners (seksmaatjes)?
☐ Nee	☐ Nee	☐ Nee
☐ Ja, met _____ eenmalige losse partner(s)	☐ Ja, met _____ meermalige losse partner(s)	☐ Ja, met _____ vaste seks partner(s) (seksmaatjes)

8. Heeft u zich door losse partners laten neuken?
☐ Nee
☐ Ja
Namelijk:

Met eenmalige losse partners?	Met meermalige losse partners?	Met vaste seks partners (seksmaatjes)?
☐ Nee	☐ Nee	☐ Nee
☐ Ja, met _____ eenmalige losse partner(s)	☐ Ja, met _____ meermalige losse partner(s)	☐ Ja, met _____ vaste seks partner(s) (seksmaatjes)
Hoe vaak heeft u daarbij een condoom gebruikt?	Hoe vaak heeft u daarbij een condoom gebruikt?	Hoe vaak heeft u daarbij een condoom gebruikt?
☐ Nooit	☐ Nooit	☐ Nooit
☐ Meestal niet	☐ Meestal niet	☐ Meestal niet
☐ Soms wel – soms niet	☐ Soms wel – soms niet	☐ Soms wel – soms niet
☐ Meestal wel	☐ Meestal wel	☐ Meestal wel
☐ Altijd	☐ Altijd	☐ Altijd

9. Heeft u zich onbeschermd laten neuken door een losse partner van wie u de HIV-status niet wist?
☐ Nee
☐ Ja.
Namelijk:

Door eenmalige losse partners?	Door meermalige losse partners?	Door vaste seks partners (seksmaatjes)?
☐ Nee	☐ Nee	☐ Nee
☐ Ja, door _____ eenmalige losse partner(s)	☐ Ja, door _____ meermalige losse partner(s)	☐ Ja, door _____ vaste seks partner(s) (seksmaatjes)

10. Heeft u zich onbeschermd laten neuken door een losse partner van wie u al wist dat hij HIV-geïnfecteerd was?
☐ Nee
☐ Ja.
Namelijk:

Door eenmalige losse partners?	Door meermalige losse partners?	Door vaste seks partners (seksmaatjes)?
☐ Nee	☐ Nee	☐ Nee
☐ Ja, door _____ eenmalige losse partner(s)	☐ Ja, door _____ meermalige losse partner(s)	☐ Ja, door _____ vaste seks partner(s) (seksmaatjes)

11. Heeft u in de afgelopen 6 maanden de volgende middelen gebruikt?
Zo ja, in welke context en op welke wijze? (Meerdere antwoorden mogelijk)

In het **algemeen**			Tijdens seks met				
		**En zo ja hoe vaak?**	**vaste partners:**	**eenmalige losse partners:**	**meermalige losse partners:**	**vaste seks partners:**	**groepsseks:**
Alcohol	☐ nee	☐ 1x per maand of minder<br>☐ 2-4x per maand<br>☐ 2-3x per week<br>☐ 4x per week of vaker	☐ ja	☐ ja	☐ ja	☐ ja	☐ ja
Poppers	☐ nee	☐ 1x per maand of minder<br>☐ 2-4x per maand<br>☐ 2-3x per week<br>☐ 4x per week of vaker	☐ ja	☐ ja	☐ ja	☐ ja	☐ ja
XTC (Ecstasy,MDMA)	☐ nee	☐ 1x per maand of minder<br>☐ 2-4x per maand<br>☐ 2-3x per week<br>☐ 4x per week of vaker	☐ ja	☐ ja	☐ ja	☐ ja	☐ ja
Erectiemiddelen (Viagra, Sildenafil, Tadalafil, Cialis, Vardenafil, Levrita, Kamagra)	☐ nee	☐ 1x per maand of minder<br>☐ 2-4x per maand<br>☐ 2-3x per week<br>☐ 4x per week of vaker	☐ ja	☐ ja	☐ ja	☐ ja	☐ ja
Hasj/Weed	☐ nee	☐ 1x per maand of minder<br>☐ 2-4x per maand<br>☐ 2-3x per week<br>☐ 4x per week of vaker	☐ ja	☐ ja	☐ ja	☐ ja	☐ ja
Cocaïne	☐ nee	☐ 1x per maand of minder<br>☐ 2-4x per maand<br>☐ 2-3x per week<br>☐ 4x per week of vaker	☐ ja	☐ ja	☐ ja	☐ ja	☐ ja
MiauwMiauw (Mephedrone, 4-MMC)	☐ nee	☐ 1x per maand of minder<br>☐ 2-4x per maand<br>☐ 2-3x per week<br>☐ 4x per week of vaker	☐ ja	☐ ja	☐ ja	☐ ja	☐ ja
GHB	☐ nee	☐ 1x per maand of minder<br>☐ 2-4x per maand<br>☐ 2-3x per week<br>☐ 4x per week of vaker	☐ ja	☐ ja	☐ ja	☐ ja	☐ ja
GBL	☐ nee	☐ 1x per maand of minder<br>☐ 2-4x per maand<br>☐ 2-3x per week<br>☐ 4x per week of vaker	☐ ja	☐ ja	☐ ja	☐ ja	☐ ja
Ketamine (Special K)	☐ nee	☐ 1x per maand of minder<br>☐ 2-4x per maand<br>☐ 2-3x per week<br>☐ 4x per week of vaker	☐ ja	☐ ja	☐ ja	☐ ja	☐ ja
Paddenstoelen	☐ nee	☐ 1x per maand of minder<br>☐ 2-4x per maand<br>☐ 2-3x per week<br>☐ 4x per week of vaker	☐ ja	☐ ja	☐ ja	☐ ja	☐ ja
Amfetamine (speed, PEP, amf, appelspeed)	☐ nee	☐ 1x per maand of minder<br>☐ 2-4x per maand<br>☐ 2-3x per week<br>☐ 4x per week of vaker	☐ ja	☐ ja	☐ ja	☐ ja	☐ ja
Methylamfetamine (perf, ice, crank, crystalmeth, yabba)	☐ nee	☐ 1x per maand of minder<br>☐ 2-4x per maand<br>☐ 2-3x per week<br>☐ 4x per week of vaker	☐ ja	☐ ja	☐ ja	☐ ja	☐ ja

	In het **algemeen**		Tijdens **seks met**				
	**En zo ja hoe vaak?**		**vaste partners:**	**eenmalige losse partners:**	**meermalige losse partners:**	**vaste seks partners:**	**groepsseks:**
Heroïne	☐ nee	☐ 1x per maand of minder<br>☐ 2-4x per maand<br>☐ 2-3x per week<br>☐ 4x per week of vaker	☐ ja	☐ ja	☐ ja	☐ ja	☐ ja
Apomorfine (Uprima)	☐ nee	☐ 1x per maand of minder<br>☐ 2-4x per maand<br>☐ 2-3x per week<br>☐ 4x per week of vaker	☐ ja	☐ ja	☐ ja	☐ ja	☐ ja
Testosteron	☐ nee	☐ 1x per maand of minder<br>☐ 2-4x per maand<br>☐ 2-3x per week<br>☐ 4x per week of vaker	☐ ja	☐ ja	☐ ja	☐ ja	☐ ja
Anabole steroïden	☐ nee	☐ 1x per maand of minder<br>☐ 2-4x per maand<br>☐ 2-3x per week<br>☐ 4x per week of vaker	☐ ja	☐ ja	☐ ja	☐ ja	☐ ja
DHEA	☐ nee	☐ 1x per maand of minder<br>☐ 2-4x per maand<br>☐ 2-3x per week<br>☐ 4x per week of vaker	☐ ja	☐ ja	☐ ja	☐ ja	☐ ja
4-FA/4-FMP	☐ nee	☐ 1x per maand of minder<br>☐ 2-4x per maand<br>☐ 2-3x per week<br>☐ 4x per week of vaker	☐ ja	☐ ja	☐ ja	☐ ja	☐ ja
2-CB	☐ nee	☐ 1x per maand of minder<br>☐ 2-4x per maand<br>☐ 2-3x per week<br>☐ 4x per week of vaker	☐ ja	☐ ja	☐ ja	☐ ja	☐ ja
Antidepressiva (niet als medicatie)	☐ nee	☐ 1x per maand of minder<br>☐ 2-4x per maand<br>☐ 2-3x per week<br>☐ 4x per week of vaker	☐ ja	☐ ja	☐ ja	☐ ja	☐ ja
Ritalin (niet als medicatie; methylphenidaat, Concerta, dexamfetamine)	☐ nee	☐ 1x per maand of minder<br>☐ 2-4x per maand<br>☐ 2-3x per week<br>☐ 4x per week of vaker	☐ ja	☐ ja	☐ ja	☐ ja	☐ ja
Opiaten (niet als medicatie)	☐ nee	☐ 1x per maand of minder<br>☐ 2-4x per maand<br>☐ 2-3x per week<br>☐ 4x per week of vaker	☐ ja	☐ ja	☐ ja	☐ ja	☐ ja
Benzodiazepinen (niet als medicatie; bijv. valium, oxazepam)	☐ nee	☐ 1x per maand of minder<br>☐ 2-4x per maand<br>☐ 2-3x per week<br>☐ 4x per week of vaker	☐ ja	☐ ja	☐ ja	☐ ja	☐ ja
Anders, nl ____________________	☐ nee	☐ 1x per maand of minder<br>☐ 2-4x per maand<br>☐ 2-3x per week<br>☐ 4x per week of vaker	☐ ja	☐ ja	☐ ja	☐ ja	☐ ja

<u>Original questions on PrEP use and intention to use PrEP (Dutch)</u>
Questionnaire wave 1

12. Heeft u ooit zelf PrEP gebruikt?
    - ☐ Nee
    - ☐ Ja

13. Heeft u in de laatste 6 maanden dagelijkse PrEP gebruikt?
    - ☐ Nee
    - ☐ Ja

14. Wanneer bent u gestart met het gebruik van dagelijkse PrEP? (Indien u meedere keren gestart bent met PrEP, vul dit dan alleen voor de laatste keer in). _ _ / _ _ _ _ (MM/JJJJ)

15. Gebruikt u nu nog steeds dagelijks PrEP?
    - ☐ Nee, ik ben gestopt op: _ _ / _ _ _ _ (MM/JJJJ)
    - ☐ Ja

16. Heeft u in de laatste 6 maanden intermitterende PrEP gebruikt?
    - ☐ Nee
    - ☐ Ja

17. Waar heeft u PrEP vandaan? (Meerdere antwoorden mogelijk)
    - ☐ Uit het buitenland
    - ☐ Via internet besteld
    - ☐ Via vrienden gekregen
    - ☐ Via iemand die hiv-positief is
    - ☐ Arts van het AmPrEP-project (soa polikliniek Amsterdam)
    - ☐ Hiv-behandelaar in het ziekenhuis
    - ☐ Anders, namelijk: ___

18. Stel dat PrEP beschikbaar komt in Nederland:
    a) Bent u dan van plan om dagelijks PrEP te gaan gebruiken?
       Zeer zeker niet 1 2 3 4 5 6 7 Zeer zeker wel
    b) Hoe waarschijnlijk is het dan dat u dagelijks PrEP gaat gebruiken?
       Heel erg waarschijnlijk 1 2 3 4 5 6 7 Heel erg onwaarschijnlijk
    c) Bent u dan van plan om intermitterende PrEP te gaan gebruiken?
       Zeer zeker niet 1 2 3 4 5 6 7 Zeer zeker wel
    d) Hoe waarschijnlijk is het dan dat u intermitterende PrEP gaat gebruiken?
       Heel erg waarschijnlijk 1 2 3 4 5 6 7 Heel erg onwaarschijnlijk

19. Stelt u zich voor dat u PrEP gaat gebruiken als dit beschikbaar komt in Nederland. Welk schema zou dan het beste bij u passen? Ga er bij het beantwoorden van de volgende vraag vanuit dat ze even effectief zijn in het voorkomen van een hiv infectie.
    - ☐ Dagelijks PrEP slikken
    - ☐ Intermitterende PrEP slikken

<u>Original questions on PrEP use (Dutch)</u>
Questionnaire wave 2-4

20. Heeft u in de laatste 6 maanden PrEP gebruikt?
    - ☐ Nee
    - ☐ Ja

21. Hoe heeft u PrEP in de laatste 6 maanden gebruikt?
    - ☐ Ik gebruik(te) PrEP elke dag
    - ☐ Ik gebruik(te) PrEP intermitterend (intermitterend betekent het niet dagelijks slikken van PrEP, maar bijvoorbeeld alleen voor en na seks waarbij ik risico op hiv had kunnen lopen)

22. Wanneer bent u gestart met het gebruik van dagelijkse PrEP? Indien u meerdere keren gestart bent met PrEP, vul dit dan alleen voor de laatste keer in.

23. Gebruikt u nu nog steeds dagelijks PrEP?
    - ☐ Ja
    - ☐ Nee, ik ben gestopt op: _ _ / _ _ _ _ (MM/JJJJ)

Hoe heeft u PrEP verkregen?
- ☐ In het buitenland gekocht
- ☐ Via internet besteld
- ☐ Van vrienden gekregen
- ☐ Van iemand die hiv-positief is gekregen
- ☐ Ik heb dit van iemand gekocht
- ☐ Ik doe mee aan het AMPrEP-project (soa polikliniek Amsterdam)
- ☐ Een hiv-behandelaar in het ziekenhuis heeft dit voorgeschreven
- ☐ Een arts anders dan een hiv-behandelaar heeft dit voorgeschreven
- ☐ Anders, nl ………………
