# Supplementary material for: Pre-exposure prophylaxis among men who have sex with men in the Amsterdam Cohort Studies: Use, eligibility, and intention to use
Source: PLoS One. 2018 Oct 12;13(10):e0205663. doi: 10.1371/journal.pone.0205663 (PMC6185853; doi:10.1371/journal.pone.0205663)
Supplement: S2 File — (PDF) [file pone.0205663.s002.pdf]

Supplement 2 Figure. Calculation of the estimated number of expected PrEP users in Amsterdam, the Netherlands.

It is estimated that there are 28,000 MSM in Amsterdam [1]. The HIV prevalence was estimated to be 13.7% (95% confidence interval (CI) 12.4 – 15.2%) in Amsterdam in 2015 [2]. Using these data, we can derive that there are 3,836 (range 3,472 – 4,256) HIV positive MSM in Amsterdam, leaving an HIV negative population of 24.164 (range 23,744 – 24,528) MSM. Based on the proportion of MSM eligible for PrEP in the Amsterdam Cohort Studies (ACS) (32.3%, 95%-CI 28.3 – 36.8%), we estimate that there are 7.805 (range 6,720 – 9,026) MSM eligible for PrEP in Amsterdam. Among eligible MSM in the ACS, 51.4% (95%-CI 43.2 – 59.5%) had a high intention and 48.6% (95%-CI 40.5 – 56.8%) a low or medium intention to use PrEP. We therefore estimate that 4,012 (range 2,903 – 5,370) MSM in Amsterdam are indicated for PrEP and are expected to use PrEP, and 3,793 (range 2,722 – 5,127) MSM are indicated, but not expected to use PrEP. Among non-eligible MSM in the ACS, 24.2% (95%-CI 19.9 – 29.1%) had a high intention to use PrEP and 75.8% (95%-CI 70.9 – 80.1%) had a low or medium intention, leading to an estimated 3,953 (range 2,986 – 5,118) MSM in Amsterdam who are not indicated for PrEP, but expected to use PrEP, and 12,382 (range 10,639 – 14,087) MSM who are not indicated and not expected to use PrEP.

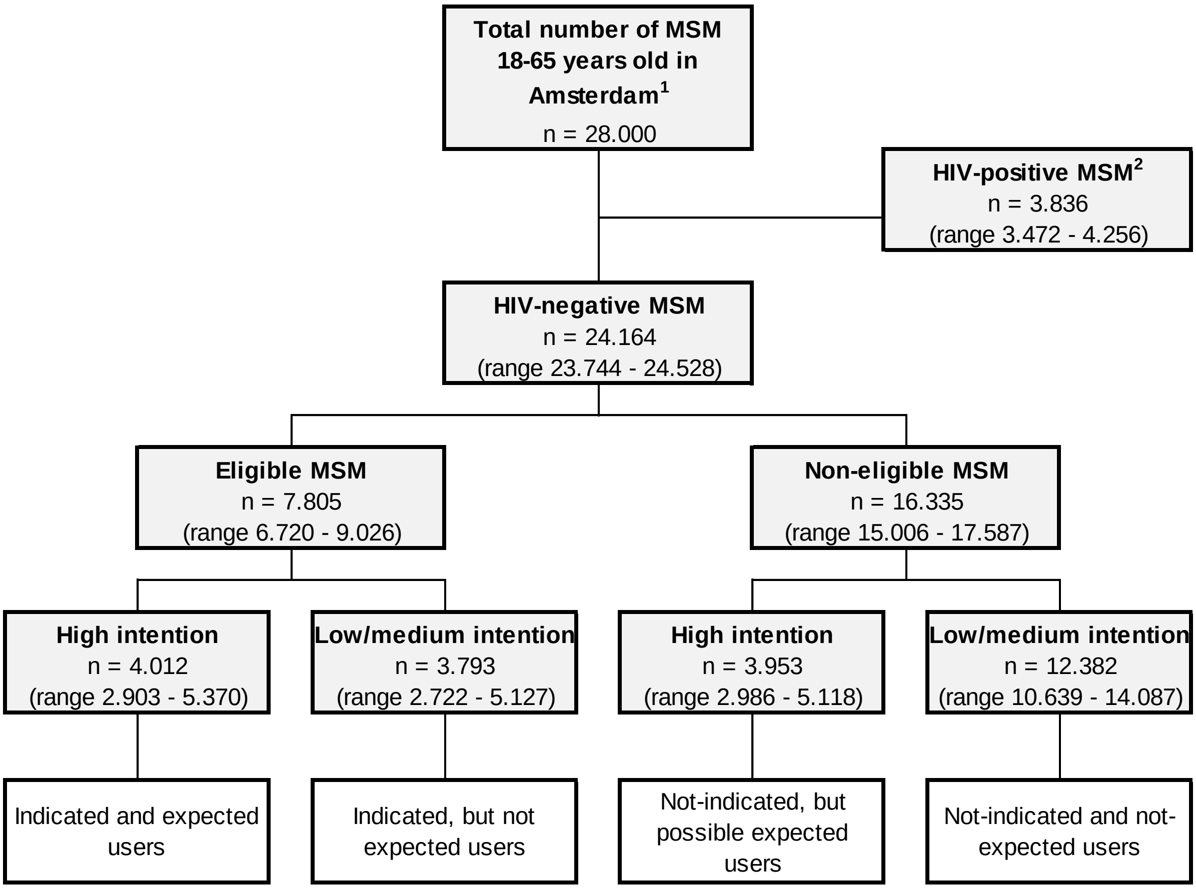

References:

1. Dijkshoorn, H., et al., *Amsterdamse Gezondheidsmonitor 2012. Online available at:* *www.ggd.amsterdam.nl/agm*. p. 123.
2. Op de Coul, E.L., et al., *Changing Patterns of Undiagnosed HIV Infection in the Netherlands: Who Benefits Most from Intensified HIV Test and Treat Policies?* PLoS One, 2015. **10**(7): p. e0133232.
